# PALB2 mutations in BRCA1/2-mutation negative breast and ovarian cancer patients from Poland

**DOI:** 10.1186/s12920-017-0251-8

**Published:** 2017-03-09

**Authors:** Anna Kluska, Aneta Balabas, Magdalena Piatkowska, Katarzyna Czarny, Katarzyna Paczkowska, Dorota Nowakowska, Michal Mikula, Jerzy Ostrowski

**Affiliations:** 1 Department of Genetics, Maria Sklodowska-Curie Memorial Cancer Center and Institute of Oncology, Roentgena 5, 02-781 Warsaw, Poland; 2 Department of Gastroenterology, Hepatology and Clinical Oncology, Medical Center for Postgraduate Education, 01-813 Warsaw, Poland

**Keywords:** Hereditary breast and ovarian cancer, PALB2, Next-generation sequencing

## Abstract

**Background:**

The *PALB2* gene encodes a protein that plays a crucial role in maintaining genomic integrity. Germline inactivating mutations in *PALB2* are associated with an increased risk of breast and ovarian cancer. The prevalence and spectrum of recurrent *PALB2* germline mutations in breast and ovarian cancer patients from Poland is not clearly defined.

**Methods:**

*PALB2* exons were amplified from 460 *BRCA1/2*-mutation negative women with familial breast and/or ovarian cancer and early-onset breast cancer using AmpliSeq technology and sequenced on an Ion Torrent PGM sequencer. In addition, eight selected variants were genotyped using TaqMan assays in 807 *BRCA1/2*-mutation negative breast cancer patients and 1690 healthy women.

**Results:**

Two recurrent *PALB2* mutations, c.172_175delTTGT and c.509_510delGA, were identified, along with one novel mutation, c.347insT. In total, *PALB2* pathogenic mutations were detected in 7/460 (1.5%) patients. Furthermore, in breast and/or ovarian cancer patients, several single nucleotide variants (SNVs) were detected in the *PALB2* coding region. In an additional group of 807 patients, eight (1%) carriers of two pathogenic mutations, c.172_175delTTGT (0.5%) and c.509_510delGA (0.5%), were identified. The c.509_510delGA mutation was not identified in healthy controls, while c.172_175delTTGT was identified in 4/1690 (0.24%) of control women.

**Conclusions:**

Germline mutations in the *PALB2* gene were observed at a frequency of approximately 1.5% in Polish breast and/or ovarian cancer patients**.** Our study confirms two recurrent *PALB2* mutations; c.172_175delGA and c.509_510delGA.

## Background

Breast cancer is the most frequently diagnosed malignancy and the leading cause of cancer death in women worldwide [1]. About 5–10% of breast and 10% of ovarian cancers are thought to be hereditary [2]. Mutations in the two main susceptibility genes, *BRCA1* and *BRCA2*, account for 20% of hereditary breast cancer (HBC) and 30% of hereditary breast and ovarian cancer (HBOC) [3]. Other genes participating in DNA-damage response pathways, including *CHEK2*, *NBS1*, *ATM*, *BRIP1*, and *PALB2*, are also involved in HBC and HBOC [4, 5].

The *PALB2* (MIM 610355, *Partner and* *Localizer of* *B* *RCA* *2*) gene was identified in a search for novel components of endogenous BRCA2 containing complexes [6]. The PALB2 protein interacts with both BRCA1 and BRCA2 through its N-terminal coiled-coil and C-terminal WD-40 domains, respectively. These three proteins form a “BRCA complex” in which PALB2 acts as a bridge between BRCA1 and BRCA2. The “BRCA complex” is crucial for initiating homologous recombination in the DNA-damage response [6–8].

Biallelic inactivating germline mutations in the *PALB2* gene lead to Fanconi anemia (Fanconi anemia type N), whereas monoallelic mutations are associated with an increased risk of breast, pancreatic, and possibly ovarian cancer [9–14]. Germline mutations in *PALB2* are responsible for 1–3.9% of HBC [15–26]. Similar to *BRCA2,* germline mutations in *PALB2* are also associated with hereditary predisposition to male breast cancer. To date, approximately 50 truncating mutations in *PALB2* have been detected in breast cancer families worldwide.

The first breast cancer family-based association study estimated that a relative risk of 2.3 (95% CI: 1.4–3.9) is conferred by mutations of *PALB2* [12]. Most recently, Antoniou and colleagues*.* applied a modified segregation procedure to show that the age-specific risk of breast cancer in female mutation carriers overlaps with that conferred by *BRCA2* mutations [27].

In a previous study, we demonstrated that in a group of 512 patients for whom targeted *BRCA1/2* mutation testing did not show any pathogenic variants, sequencing of *BRCA1/2* exons identified 52 carriers of mutations [28, 29]. The aim of the present study was to investigate the contribution of *PALB2* germline mutations in a group of 460 *BRCA1/2*-mutation negative breast and/or ovarian cancer patients and identify the optimal panel of recurrent mutations for genetic screening of *PALB2*.

## Methods

### Patients

DNA samples were chosen from the repository of the Genetic Counseling Unit, Maria Sklodowska-Curie Memorial Cancer Center and Institute of Oncology in Warsaw. In-depth interviews assessed personal and familial cancer history. Healthy women with no known history of cancer were enrolled from the National Colorectal Cancer Screening Program. Patients comprised a group of 460 *BRCA1/2*-mutation negative breast cancer patients (with a median age of 43 years, range 17–68 years) and included 165, 103, and 192 women with HBC, HBOC, and early-onset breast cancer (EOBC), respectively [29]. This group of patients had undergone full *BRCA1/2* sequencing using the Ion AmpliSeq BRCA1 and BRCA2 Panel (Thermo) on a PGM sequencer as part of our previous study [29].

An additional group, comprising 807 selected *BRCA1/2*-mutation negative women, was surveyed using TaqMan SNP genotyping assays. This group had a median age of 48 years (range 20–85 years); included 322, 81, and 404 HBC, HBOC, and EOBC patients, respectively; and were negative for 20 selected *BRCA1/2* mutations, including 11 mutations in *BRCA1*, namely, c.66_67delAG, c.181T > C, c.3756delGTCT, c.3700_3704del5, c.4035delA, c.3777delT, c.4065delTCAA, c.4041delAG, c.5263delC, c.5213G > A, and 5370C > T, and nine mutations in *BRCA2*, namely, c.1408G > T, c.5946delT, c.5239insT, c.6447delTA, c.5964delAT, c.7910del5, c.9382C > T, c.8924delT, and c.9402delT [28].

### NGS of PALB2

Genomic DNA was extracted from peripheral blood, as previously described [28]. DNA concentrations were determined using a Qubit 2.0 Fluorometer (Thermo) and the dsDNA HS Assay Kit (Thermo). For library construction, the Ion AmpliSeq™ Library Kit 2.0 (Thermo) and Ion AmpliSeq *PALB2* custom primers, comprising 27 primer pairs spanning 5.29 kb, were used to amplify the coding regions of the *PALB2* gene. Library concentrations and size distributions were determined with the Qubit dsDNA HS Assay Kit (Thermo) and a High Sensitivity DNA Analysis Kit on a Bioanalyzer 2100 (Agilent), respectively.

Up to 48 libraries were combined in equimolar concentrations following their loading onto an Ion Chef for DNA template preparation with a set of reagents from an Ion PGM IC 200 Kit and an Ion 316 Chip Kit v2 BC (Thermo). Sequencing was performed on the Ion Torrent PGM in a mode with 500-flow runs. Data were analyzed on the Ion Torrent server using the Torrent Suite software (Thermo). Sequencing reads were aligned to the hg19 reference genome and fitted to *PALB2* designed amplicons. The coverage analysis and variant caller plug-ins were run with optimal settings to identify germline variants. *PALB2* gene variants were annotated using data from ClinVar-NCBI and LOVD.

### TaqMan genotyping

Mutations found with NGS were validated by Sanger sequencing. Furthermore, eight selected variants, namely 172_175del4, c.347insT, c.509_510delGA, Gln559Arg, Glu672Gln, Leu939Trp, Gly998Glu, and Thr1100Thr were analyzed using TaqMan SNP Genotyping Assays (Thermo) on an ABI 7900HT qPCR system (Thermo), as previously described [28].

## Results

### NGS analysis

We evaluated the frequencies of *PALB2* germline mutations in 460 *BRCA1/2*-mutation negative breast and/or ovarian cancer patients. Of the 460 women, 268 had HBC and/or HBOC, and 192 had EOBC, with a median age at breast cancer diagnosis of 43 years (range 17–68 years).

In this group, we identified seven unrelated carriers (1.5%) of the three *PALB2* truncating mutations: c.172_175delTTGT (*n* = 4), c.347insT (*n* = 1), and c.509_510delGA (*n* = 2) (Table 1). Six of the seven carriers (2.2%) were among the 268 HBOC patients, and one (0.5%) was in the EOBC group.

**Table 1:** Truncating and putative pathogenic missense mutations in the *PALB2* gene revealed by NGS of samples from 460 breast cancer patients

Exon	*PALB2* cDNA change	Protein change	Age at diagnosis (years)	Family history
3	c.172_175delTTGT	p.Gln60Argfs	BR62	I- BR63, II- BR70
			BR48	I- BR58
			BR30	I- GC68, II- THYR60
			BR40	I- BR50, MEL60, PR85
4	c.347insT	p.Leu116fs	BR40	Not reported
	c.509_510delGA	p.Arg170Ilefs	BR59	I- BR66, II- BR40
			BR32	I- BR38

I and II indicate first and second degree relatives, respectively; *BR* breast cancer, *GC* gastric cancer, *THYR* thyroid cancer, *MEL* melanoma, *PR* prostate cancer

In addition to these pathogenic mutations, we detected 10 SNVs in the coding region of the *PALB2* gene (Table 2).

**Table 2:** *PALB2* genetic variants in familial breast and/or ovarian cancer and in non-familial breast cancer patients identified by NGS and TaqMan genotyping

				NGS			Taqman genotyping			
Localization	*PALB2* cDNA change	PALB2 protein change	rsID	Familial cancer (*n* = 268)	Non-familial cancer (*n* = 192)	All (*n* = 460)	Familial cancer (*n* = 403)	Non-familial cancer (*n* = 404)	All (*n* = 807)	Healthy controls (*n* = 1690)
Exon 3	c.172_175delTTGT	p.Gln60Argfs	180177143	3 (1.1%)	1 (0.5%)	4 (0.9%)	1 (0.2%)	3 (0.7%)	4 (0.5%)	4 (0.2%)
Exon 4	c.347insT	p.Leu116fs	novel	1 (0.4%)	0	1 (0.2%)	0	0	0	0
	c.509_510delGA	p.Arg170Ilefs	515726123	2 (0.7%)	0	2 (0.4%)	4 (1.0%)	0	4 (0.5%)	0
	c.1010T > C	p.Leu337Ser	45494092	8 (3.0%)	6 (3.1%)	14 (3.0%)	nt	nt	nt	nt
	c.1676A > G	p.Gln559Arg	152451	60 (22.4%)	39 (20.3%)	94 (20.4%)	101 (25%)	101 (25%)	202 (0.25%)	350 (21.3%)
	c.1572A > G	p.Ser524Ser	45472400	1 (0.4%)	0	1 (0.2%)	nt	nt	nt	nt
Exon 5	c.2014G > C	p.Glu672Gln	45532440	16 (6.0%)	7 (3.6%)	22 (4.8%)	27 (6.7%)	32 (7.9%)	59 (7.3%)	108 (6.5%)
Exon 7	c.2590C > T	p.Pro854Ser	45568339	5 (1.9%)	1 (0.5%)	6 (1.3%)	nt	nt	nt	nt
Exon 8	c.2794G > A	p.Val932Met	45624036	6 (2.2%)	1 (0.5%)	2 (0.4%)	nt	nt	nt	nt
	c.2816T > G	p.Leu939Trp	4548192	1 (0.4%)	1 (0.5%)	2 (0.4%)	4 (1.0%)	2 (0.5%)	6 (0.7%)	3 (0.18%)
Exon 9	c.2869A > G	p.Lys957Gln	515726103	0	1 (0.5%)	1 (0.2%)	nt	nt	nt	nt
	c.2993G > A	p.Gly998Glu	45551636	8 (3.0%)	7 (3.6%)	15 (3.3%)	21 (5.2%)	19 (4.7%)	40 (5.0%)	73 (4.5%)
Exon 12	c.3300T > G	p.Thr1100Thr	45516100	15 (5.6%)	7 (3.6%)	22 (4.8%)	27 (6.7%)	32 (7.9%)	59 (7.3%)	108 (6.6%)

*nt* not tested

### Genotyping of selected variants detected by NGS

Using TaqMan SNP genotyping assays, the frequencies of eight selected variants, including three truncating mutations (c.172_175delTTGT, c.347insT, c.509_510delGA), a rare missense variant (Leu939Trp), and four common polymorphisms (Gln559Arg, Glu672Gln, Gly998Glu, Thr1100Thr), were tested in an additional group of 807 breast and/or ovarian cancer patients (median age, 48 years; range 20–85 years), and 1690 healthy women (median age, 58 years; range 26–79 years).

The pathogenic mutation, c.172_175delTTGT, was identified in 4/807 (0.5%) breast cancer patients and in 4/1690 (0.24%) healthy controls (Table 2). The c.509_510delGA mutation was detected in four breast cancer patients, and was not found in healthy control subjects. The c.347insT mutation was identified in neither additional cancer patients nor healthy controls.

The frequencies of the four common polymorphisms were similar in cancer patients and healthy controls; therefore, the clinical significance of these polymorphic variants could not be confirmed. In addition, the Leu939Trp missense variant was detected in six of 807 (0.7%) cancer patients and three of 1690 (0.2%) healthy controls; hence, the incidence of this change was not significantly different between patients and controls.

## Discussion

We identified three pathogenic *PALB2* mutations among 460 *BRCA1/2*-mutation negative breast and/or ovarian cancer patients. The two recurrent mutations identified in our study were c.172_175delTTGT and c.509_510delGA. The novel pathogenic mutation c.347insT was found only in one young patient with HBC. The two recurrent mutations were identified in five patients with HBC and one patient with EOBC, but without any family history. In the cancer patient group, the recurrent variants (c.172_175delTTGT and c.509_510delGA) accounted for 86% (6/7) of *PALB2* mutations. Our NGS and TaqMan genotyping studies indicate that the pathogenic c.509_510delGA mutation contributes to familial breast and/or ovarian cancer (0.9%); however, the c.172_175delTTGT mutation was identified both in familial (0.6%) and non-familial cancer patients (0.7%). The recurrent mutation c.509_510delGA is a European founder mutation that has been identified in breast cancer patients from Poland, Belarus, Russia, and Germany [16, 17, 24, 25]. This mutation has been detected in 0.6–1.4% of breast cancer patients in three independent studies in Poland [5, 16, 26]. The other recurrent *PALB2* mutation, c.172_175delTTGT, was identified in breast cancer patients from the Czech Republic and Poland [5, 18, 26].

The *PALB2* gene mutation rate (1.5%) determined for our group of breast and/or ovarian cancer patients was similar to those reported by other studies. *PALB2* germline mutations have been identified in Finland, Germany, Poland, Czech Republic, Russia, Italy, Spain, Australia, United Kingdom, France, Netherlands, Canada, USA, China, and South Africa, with rates ranging from 0.1 to 3.9%, depending on the population [15–27]. In breast cancer families with both female and male breast cancer, *PALB2* mutation rates increased to 6.7% in a UK study and to 9.0% in a Spanish study [12, 15].

In addition to clearly pathogenic mutations, we detected 10 missense variants in the coding region of the *PALB2* gene. Identifying variants of unknown significance in cancer susceptibility genes represents a real problem in genetic counseling [30]. It should be stressed that, to date, no *PALB2* missense variant has been classified as definitely pathogenic in any large population study [31]. Park et al. reported that the rare missense variant, Leu939Trp, is associated with altered direct binding of PALB2 to the RAD51C, RAD51, and BRCA2 proteins [32]. Functionally, the Leu939Trp mutant displays a decreased capacity for DNA double-strand break repair and an increased cellular sensitivity to ionizing radiation. In our group of breast and/or ovarian cancer patients, the missense variant, Leu939Trp, was detected in two (0.4%) breast cancer patients, one (0.4%) patient with HBOC, and in one (0.5%) with EOBC. Interestingly, in our study, the missense variant rate was 3.5 times more frequent in breast cancer patients (0.7%) than in healthy controls (0.2%), although the differences were not statistically significant. Consistent with this observation, a recent large case-control study by Southey et al. found no evidence of association of the Lys939Trp variant with breast cancer risk [33]. Furthermore, Catucci and colleagues [34] have also recently shown that the Lys939Trp variant does not disrupt the homologous recombination-mediated DNA repair activity of *PALB2* and they concluded that this variant should be regarded as neutral with no clinical relevance to risk of breast cancer.

## Conclusions

In summary, our study confirmed two *PALB2* recurrent mutations, c.172_175delGA and c.509_510delGA, in *BRCA1/2*-mutation negative breast and/or ovarian cancer patients from Poland. The c.509_510delGA mutation contributed to familial cancer but was absent in non-familial cancer patients, while the c.172_175delTTGT mutation was identified in both familial and non-familial cancer patients.

## Abbreviations

BRCA1: Breast cancer 1, early onset
BRCA2: Breast cancer 2, early onset
EOBC: Early-onset breast cancer
HBC: Hereditary breast cancer
HBOC: Hereditary breast and ovarian cancer
NGS: Next-generation sequencing
